# Vision on gyrate atrophy: why treat the liver?

**DOI:** 10.1038/s44321-023-00002-0

**Published:** 2023-12-14

**Authors:** Iolanda Boffa, Nicola Brunetti-Pierri

**Affiliations:** 1 Telethon Institute of Genetics and Medicine (TIGEM), Pozzuoli, Italy; 2 Department of Translational Medicine, “Federico II” University, Naples, Italy; 3 Scuola Superiore Meridionale (SSM, School of Advanced Studies), Genomics and Experimental Medicine Program, University of Naples Federico II, Naples, Italy

**Keywords:** Genetics, Gene Therapy & Genetic Disease

## Abstract

In this Correspondence, N. Brunetti-Pierri and I. Boffa argue that liver-directed gene therapy is the preferred option for treatment of gyrate atrophy of the choroid and retina.

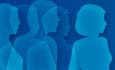

## Teaser

Gyrate Atrophy of the Choroid and Retina (GACR) currently lacks any effective treatment and a highly restricted, yet cumbersome, low-arginine diet is the only available therapeutic option. In our work, we found that replacement of ornithine-aminotransferase (OAT) activity in the liver lowers ornithine concentrations in the blood and the eye, thus improving retinal structure and function (Boffa et al, 2023).

Because a deficiency of liver OAT activity is primarily responsible for the increased systemic concentrations of ornithine, and reduced blood ornithine prevents retinal degeneration in mice and humans (Kaiser-Kupfer et al, 2002; Wang et al, 2000), the liver is the most logical target tissue for GACR gene therapy. Nevertheless, Bergen and colleagues raised concerns about the liver-directed approach for GACR based on disease mechanisms, patient’s needs, therapeutic developmental timelines, and even ethical considerations. These arguments led them to conclude that eye gene therapy might be a better therapeutic option for GACR. While we appreciate the interest raised by our study, we argue that the lack of any in vivo proof-of-concept study supporting the efficacy of retinal gene therapy for GACR makes such a discussion premature. Once subretinal injections in mouse models of GACR are proven to be as effective as liver-directed gene therapy, a careful evaluation of the risks and benefits of both approaches could be initiated. It is very possible that retinal cells with restored OAT activity could be damaged by the high systemic ornithine concentrations, thus making retinal gene transfer futile. This concern is indeed supported by findings from Bergen et al, who showed that retinal damage is fueled for the most part by vascular contribution (Bergen et al, 2019). The same concern for GACR therapy is also valid for other localized approaches, such as subretinal transplantation of retinal pigment epithelium (RPE) cells that has been performed in Stargardt macular dystrophy and age-related macular degeneration (Schwartz et al, 2015).

Assuming that it will be proven to be as effective as the liver-directed gene therapy approach for GACR, eye gene therapy would still require surgical intervention to inject the vector into the subretinal spaces of both eyes (one eye at a time). Compared to subretinal injections, the intravenous injections required for the liver-directed gene transfer are much simpler and less invasive. Subretinal injections induce iatrogenic macular detachment, although they have rarely been found to compromise retinal function. However, studies in inherited retinopathies might have overlooked the risks of subretinal injections because any visual decline induced by the iatrogenic detachment may be defrayed by improvements resulting from retinal gene transfer. Moreover, central retinal atrophy has been noted in approximately 50% of patients with Leber congenital amaurosis following iatrogenic detachment (Boye et al, 2013). In addition, risks of subretinal injections include ocular inflammation, elevated intraocular pressure, cataracts, and intraoperative retinal tears (Russell et al, 2017).

Subretinal injections of AAV result in gene transfer to an area restricted to a “ring” around the injection site, leaving the remaining cells of the retina uncorrected. In contrast, by reducing systemic hyperornithinemia, liver-directed gene therapy results in broader retinal correction and thus, greater clinical benefit. Nevertheless, we recognize that because cells in the retina express OAT, it is possible that full correction might not be achieved by reducing systemic ornithine concentrations. Under such circumstances, a combination of eye and liver gene therapy would be expected to be required for complete restoration of retinal function.

Surprisingly, Bergen and colleagues raised the issues of patients’ needs and ethical concerns about delivering a gene to an unaffected organ to achieve a therapeutic effect in another site (i.e., the eye). Based on current knowledge, gene transfer to liver cells is the only known effective strategy for the therapy of GACR and it addresses the main unmet need of vision preservation in GACR patients (Schultink, 2023). We would also underscore that no ethical concerns have been raised by the treatment of an organ that does not show clinical signs of disease itself (e.g., the liver) when therapy is designed to treat other damaged organs or body districts. Indeed, it has long been recognized that the most attractive disease candidates for liver-directed gene therapy are disorders with unaffected hepatic architecture, such as Crigler–Najjar syndrome (D’Antiga et al, 2023), some urea cycle disorders (Duff et al, 2023), and hemophilia A and B for which liver-directed gene therapies have been recently approved by the EMA and the FDA.

In clinical trials, liver-directed AAV-based approaches resulted in multi-year expression of the therapeutic gene in several disorders (Piccolo et al, 2020). Bergen and colleagues included spinal muscular atrophy among the targets of liver gene transfer. However, the liver is not the target tissue in spinal muscular atrophy, but it rather acts as a sink for systemically delivered AAV vectors resulting in hepatotoxicity (Duan, 2023). Luckily, AAV vector doses required for liver gene transfer that are sufficient to correct several inherited metabolic diseases, including GACR (Boffa et al, 2023) and hemophilia, do not result in major toxicity. Nevertheless, it should be emphasized that AAV vectors have other limitations including neutralizing antibodies, vector dilution with ongoing liver growth, and potentially long-term risks of malignancies (Costa-Verdera et al, 2023; Mucke et al, 2023). These limitations might be addressed in the future by improved AAV vectors, or better viral or nonviral gene transfer vectors. Despite these issues, AAV vectors have demonstrated remarkable clinical success and remain the most attractive vectors for liver-directed gene therapy.

In conclusion, we believe that the liver remains the optimal target tissue for the correction of the retina in GACR, and potentially also other suspected disease complications affecting the skeletal muscle and the brain (Heinanen et al, 1999; Valtonen et al, 1999) that might be secondary to the hyperornithinemia. Nevertheless, before the clinical translation of liver-directed gene transfer can be undertaken, further research is needed to address important safety concerns. Does ectopic OAT expression in periportal hepatocytes negatively affect ureagenesis? Given that OAT supports polyamine synthesis required for tumor growth (Lee et al, 2023) and its overexpression in hepatocellular carcinoma (Colnot et al, 2004), is OAT overexpression or misexpression a risk factor for liver cancer? Addressing these issues in preclinical models will provide a comprehensive risk-benefit assessment and recommendations for clinical translation of AAV-mediated liver-directed gene therapy.
